# Response of Polypropylene Composites Reinforced with Natural Fibers: Impact Strength and Water-Uptake Behaviors

**DOI:** 10.3390/polym15040900

**Published:** 2023-02-11

**Authors:** María E. Vallejos, Fabiola Vilaseca, José A. Méndez, Francisco X. Espinach, Roberto J. Aguado, Marc Delgado-Aguilar, Pere Mutjé

**Affiliations:** 1 LEPAMAP-PRODIS Research Group, Department of Chemical Engineering, Department of Organization, Business Management and Product Design, University of Girona, C/Maria Aurèlia Capmany, 61, 17003 Girona, Spain; 2 Instituto de Materiales de Misiones (IMAM), Universidad Nacional de Misiones—Consejo Nacional de Investigaciones Científicas y Técnicas (UNaM—CONICET), Posadas 3300, Argentina; 3 Advanced Biomaterials and Nanotechnology (BIMATEC), Department of Chemical Engineering, University of Girona, C/Maria Aurèlia Capmany, 61, 17003 Girona, Spain

**Keywords:** hemp strands, composites, impact strength, interface, water uptake, natural fibers, polypropylene

## Abstract

Composites from polypropylene (PP) reinforced with hemp strands (HS) are prepared in the current work with the aim of deepening on the influence of this reinforcement on the impact performance of these specific composites. Despite all the research conducted in this field, the effect of this natural reinforcement on the absorbed energy during crack formation and propagation is not fully tackled in previous research works. From the methodology and samples’ geometry, the results concluded that the quality of the interface has a noticeable role in the impact resistance of these materials. The interface strength, fiber dispersion and fiber pullout are the main contributors to crack formation, whereas fiber pullout is the main one responsible for crack propagation. Maximum values of absorbed energy were found for PP composites comprising 20–30 wt% of HS and 8 wt% of the coupling agent for the un-notched samples, whereas maximum absorbed energy values corresponded to PP composites with 40 wt% of HS and 4 wt% of coupling agent for the notched samples. The water-absorption behavior in different humid environments is also examined. From the kinetic study, the water diffusion followed a Fickean behavior showing low-diffusion coefficients, increasing with fiber content. This systematic investigation represents a contribution to the analysis of the potential of reinforcing conventional polymers with natural materials, as a strategy towards more sustainable development.

## 1. Introduction

In the current situation of climate change and global warming, aggravated in some regions by sociopolitical circumstances, the rational use of both natural and fossil resources is of great importance. The population is exponentially growing, and so is the need to guarantee sustainable development based on green principles, which implies not only using natural resources but also the reasonable consumption of fossil reserves. The so-called circular economy requires a combination of good practices starting from the resources, manufacturing, product’s lifetime, recycling, and reincorporation of materials into the loop for waste minimization [1]. A more circular plastics economy seeks to minimize the need for virgin material and energy in the production of plastics, while reducing the environmental issues linked to resource extraction, production, consumption, and waste generation. The plastic industry is dependent on fossil fuels in various ways that result in strong “carbon lock-in” throughout the value chain and large and growing CO_2_ emissions [2]. The 2022 United Nations Environment Assembly resolution for a global agreement on plastic pollution shows that the issue is now on the top of the environmental governance agenda, after repeated calls from researchers. In this context, fossil resources in the form of polymer commodities, such as polypropylene (PP), are expected to be employed in a more rational approach [2].

It is known that reinforcing conventional polymers with natural materials is one strategy towards the envisioned sustainable development. Hemp and lignocellulosic hemp derivatives fall into this category [3,4,5,6,7]. The authors have large experience in the full exploitation of hemp as reinforcement/filler of thermoplastic composite materials [8]. Studies were conducted on the response to the tensile properties of the micromechanics of hemp strands PP composites and also on the use and influence of coupling agents [9]. The tensile and flexural properties of hemp strands-PP composites were later investigated after treatment with different concentrations of NaOH [10]. Since the intrinsic properties of the reinforcing elements are one key parameter on the final properties of composites, the inherent characteristics of hemp strands, as well as the interface quality, were thoroughly studied in specific PP/hemp strand composites [11]. Other conventional polymers or biobased polymers, such as thermoplastic starch [12], were used as matrices of hemp lignocellulosic derivatives, and their mechanical and thermal response was analyzed. Other works are found in the literature for polymer-based composites reinforced with hemp fibers and hemp strands. Pracella et al. [13] studied the general functionalization and compatibilization of hemp fibers with PP. In 2016, Ngaowthong et al. [14] showed the morphology, mechanical, thermal properties, and water absorption behaviors of PP/hemp woody core fiber composites. The behavior of hemp textile fibers as PP reinforcement has also been investigated [15,16]. The thermal stability of PP-hemp composites [17] and the properties of PP/hemp fibers flame-retardant composites [18] were analyzed, with the incorporation of ammonium polyphosphate and organomodified montmorillonite. The influence of the processing temperatures on the microstructure of PP/hemp fiber composites was studied by different authors [19,20]. Producing hybrid composites from hemp and recycled carbon fiber has been used as a strategy to improve composite performance and broaden the range of applications while using low-cost processing [21,22]. Hemp fibers were also submitted to different treatments and modifications to improve the final composite performance [23,24,25,26]. Other interesting works on the crystallization behavior were conducted by Niu [27] or Hargitai [28].

Despite all these research efforts, little attention is still paid to how the impact properties are affected by the use of hemp fibers or hemp strands. Very often, the composite performance requires a combination of properties in static and dynamic conditions. Even though composites show more improved modulus than the plain matrix, the strength properties are very much affected by the interface quality and, as a result, the composite resistance might decrease with the fiber content. This phenomenon is more apparent when impact strengths are considered. Systematic investigation of composite impact resistance is a complicated challenge since the result of an impact test is not solely dependent on material parameters but also a test configuration and sample configuration [29]. The current investigation aims to deepen the effect of hemp strands on the impact performance of their PP composites. Similarly, the influence of hemp strands on the water uptake of these PP composites is another concern of the current investigation. The response of PP composites reinforced with hemp strands under impact conditions and different relative humidity environments are examined and discussed in this work.

## 2. Materials and Methods

### 2.1. Materials

Hemps strands containing around 25% of straw were supplied by Agrofibra A.L. (Puigreig, Spain). The initial length (20–30 cm) was chopped by means of a blade mill using a 10 mm sieve. Hemp straw was removed in a flotation cell (1% consistency) equipped with a low rotor speed (500 rpm) for 20 min. Hemp bundles were dried in an oven at 80 °C for 24 h before use. PP homopolymer from Repsol Chemicals (Madrid, Spain) was used as a thermoplastic matrix. Isplen PP 090 G2M injection grade with a melt flow index of 30 g/10 min was used for the composite preparation. In order to improve the fiber-matrix interface adhesion and compatibility, maleated-polypropylene Epolene G 3015 supplied by Eastmann España S.L. (Sant Celoni, Spain) was used in each formulation. Finally, xylene from Sigma Aldrich Chemie was used as a PP solvent.

### 2.2. Methods

#### 2.2.1. Preparation of PP-Hemp Strands Composites

Composite blends from PP comprising 20, 30, and 40 wt% of hemp strands were prepared using a roll mixer from IQAP LAB S.L. (Roda de Ter, Spain) working at 180 ± 5 °C for about 10 min at about 50 rpm. For each formulation, different amounts of coupling agent were tested, in particular, 0, 2, 4, 6, and 8 wt%, concerning the reinforcement content. The composite blends were cooled down and pelletized in a blade mill from Agrimsa (St. Adrià del Besós, Spain) provided with a 100 sieve. Afterward, the composite pellets were molded using an injection molding machine Mateu&Soler-35T (Barcelona, Spain). This injection machine is equipped with a closing pressure of 40 Tn and three heating areas were stabilized at 180 °C, 190 °C, and 210 °C, being the highest that closer to the nozzle. The first and second injection pressures were 120 and 25 kgf cm^−2^, respectively. The steel mold used was built according to ASTM D3641. Prior to testing, the samples were conditioned at 80 °C for 48 h at 50% relative humidity, according to ASTMD618 standard. The measured properties were compared to those of a plain polymer matrix.

**Figure 1:**
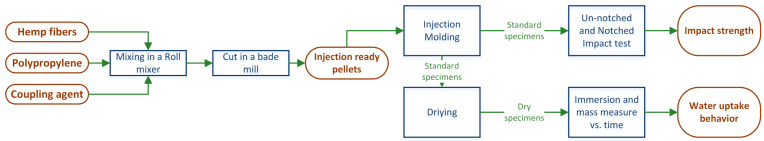
Flow-chart of the experimental methods in the current investigation.

The flow-chart scheme for the experimental procedure of the current work is shown in Figure 1.

#### 2.2.2. Characterization of the Impact Properties

Impact properties from both Charpy and Izod methods were considered in the current work. Charpy impact tests were performed from notched and un-notched specimens by means of the Resil 5,5 hammer from Ceast Instruments (Pianezza, Italy). Standard ISO 179 was followed for this test. The absorbed energy for crack formation and fracture propagation was determined from the un-notched and notched samples. The impact resistance of materials was also determined following the Izod methodology according to ISO 180 standards. The energy absorbed by the sample is calculated from the height the arm swings to after hitting the sample. In the Izod test, a notched sample is generally used to determine impact energy and notch sensitivity. At least five specimens were tested in every case. Standard error and the significant features were based on the rules proposed by Taylor [30].

#### 2.2.3. Water-Uptake Analysis

The water uptake of composites was measured from two different tests, using a Dycometal climatic chamber (Viladecans, Spain) and by water immersion. Composite specimens were dried at 105 °C for 2 h to remove any residual moisture before starting any analysis. After drying, samples were placed in the Dycometal climatic chamber working at 23 °C and 50% relative humidity. Samples were weighted at determined times and the water uptake was measured. In the immersion test, after drying, the samples were immersed in distilled water at 23 °C. Samples were kept under immersion until reaching equilibrium. The water uptake was calculated by the difference in weight (*W*) from the initial weight (*W*_0_) of the samples (Equation (1)). Water uptake at the equilibrium state (*W_∞_*) was determined when the constant weight of samples was achieved.

(1) $$Water\;uptake\;\left(\%\right)=\frac{W-W_{0}}{W_{0}}\times 100$$

## 3. Results and Discussion

### 3.1. Impact Strength of PP/HS Composites

Composite specimens with hemp strands (HS) and maleated polypropylene (MAPP) contents ranging from 20 to 40 wt% and 0 to 8 wt%, respectively, were submitted to un-notched and notched Charpy impact tests and to the Izod impact test. Table 1 shows the experimental results.

The obtained results allowed us to analyze the effect of the hemp strands reinforcing elements and of the MAPP coupling agent on the impact properties of the PP composites. Having un-notched and notched results evaluates the energy devoted to crack creation and propagation possible.

**Figure 2:**
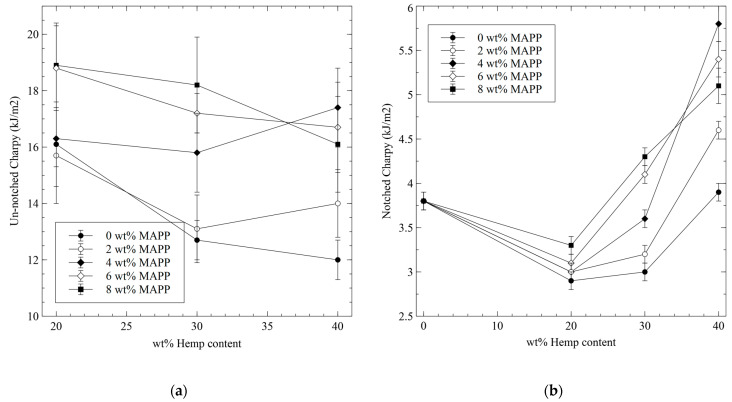
Evolution of the impact strength against MAPP and HS contents: (**a**) for un-notched Charpy specimens; (**b**). for notched Charpy specimens.

The equipment used for the un-notched tests was unable to break the PP specimen. This is due to the dimensions of the equipment with a hammer of 2.074 kg and a length of 380 mm, unable to generate enough energy. The rest of the un-notched specimens broke under impact conditions. The results show that both, reinforcement and coupling agent contents, had an impact on the strength of the composites. The evolution of the un-notched impact strengths against HS and MAPP contents is presented in Figure 2a.

Composites of 0 and 2 wt% MAPP content showed a decrease in their impact strength with HS content. This can indicate the propagation of the fracture through the interface due to poor adhesion between the fibers and the matrix [29]. This can be expected in the case of natural fibers as a polyolefin reinforcement, due to the different polarity of the phases. On the one hand, natural fibers are hydrophilic, and on the other hand, PP is hydrophobic [31]. The impact strength for the composites at 20 wt% HS and 0 to 4 wt% MAPP contents were almost the same, indicating that for such HS contents, MAPP showed a limited effect. Uncoupled composites decreased their impact strength noticeably with increasing HS contents. This indicates a poor or null contribution of the reinforcements to the impact strength of the composites. In this case, the impact strength of the composites depends on the impact strength of the matrix. Another explanation reported in the literature is the agglomeration of fibers due to poor dispersion that creates areas with stress concentration that need less energy to propagate a fracture [32,33]. A weak interface along with fiber agglomerations will produce non-uniform stress transfer and this phenomenon will increase with increasing fiber contents [34,35]. These stress concentration areas will increase with increasing reinforcement contents. In the case of the composite with a 2 wt% of MAPP, the evolution is like the uncoupled composites for 20 to 30 wt% HS contents. In the case of the composite with 40 wt% HS content, the impact strength shows a tendency to increase. This can be because MAPP content is evaluated against HS content, and thus, the composite with 40 wt% HS has more MAPP w/w than its 30 wt% HS counterparts. This change in the coupling agent content can be enough to increase the strength of the interface and allow the propagation of some of the impact energy to the fibers. Moreover, adding MAPP increases the wettability of the fibers and promotes a better dispersion of the fiber preventing agglomerations. The composite that added 4 wt% of MAPP showed a tendency to increase its impact strength against HS content. Composites with 20 and 30 wt% HS contents showed almost the same impact strength, and the composite adding 40 wt% of HS increased noticeably its strength. This can be explained similarly to the case of the composites with 2 wt% of MAPP. The increasing presence of MAPP increased the strength of the interface and allowed transferring energy from the matrix to the reinforcements. Moreover, the interface is not strong enough to retain the fibers and some energy is devoted to fiber pullout during impact break, increasing the amount of energy needed to propagate the fracture. Composite materials with 6 and 8 wt% MAPP contents showed similar impact strength for 20 wt% HS contents. Such impact strengths were noticeably higher than those of composites with lesser MAPP contents. Thus, for low HS contents, the amount of coupling agent needed to obtain interfaces strong enough to affect the impact properties is higher. This is also related to the method used by the researchers to compute the coupling agent percentage against HS content. Higher HS contents decreased the impact strength of the composites. In the case of the 6 wt% MAPP composite, the regression curve seem to stabilize, but in the case of the 8 wt%, MAPP content the curve shows a tendency to descent. A strong interface can reduce the deformability of the material and increase its fragility, this phenomenon increases with increasing percentages of reinforcement [36]. Some authors announce that high percentages of coupling agents can promote self-entanglement between the compatibilizer and cause a descent of the impact strength [37]. Moreover, the impact strength of the composite with 4 wt% MAPP and 40 wt% HS was higher than its counterparts. This can be due to an interface strong enough to retain the fibers and not allow fiber pullout. In the case of composites with MAPP contents higher than 4 wt%, such an interface can be strong enough to allow fracture propagation through the fiber, decreasing the fracture area and precluding fiber pullout phenomena.

In all cases, the impact strength of the composites was lower than the matrix. This was expected as the matrix is a ductile material and the composites are fragile. The decrease in the impact strength of un-notched composites can be related to a change from ductile to brittle fracture. The same composites, submitted to tensile strength showed an increase in fragility, and a decrease of their strain at the break when the tensile was tested [38].

The analysis shows that the best coupling agent contents to optimize the impact strength of the composites vary with the amount of reinforcement. For composites with 20 to 30 wt% HS contents, 8 wt% MAPP returned the highest values. For composites with 40 wt% HS content, 4 wt% MAPP showed the highest results. MAPP contents have a major influence over the strength of the interface and thus over the tensile and flexural strength. Hence, during the formulation of the composite materials, the properties to optimize and the effect of such optimization over other properties has to be taken into account. Nurbakhsh and Ashori [31] advised on the necessity of establishing an optimum interfacial adhesion to obtain good impact strengths for natural fiber-reinforced composites. Despite the tendency of the regression curve, it must be taken into account that some researchers remark on the existence of optimum reinforcement percentages above which the impact strength decreases [39,40,41].

Notched specimens submitted to the Charpy test showed a noticeable decrease in the values regarding the un-notched specimens (Table 1). The values show that impact strength is noticeably impacted by the percentage of HS, and also by the presence and content of the coupling agent. Figure 2b shows the evolution of the notched Charpy impact strength of the specimens against HS and MAPP contents.

The equipment was able to break the PP-notched specimen, with an impact strength of 3.8 ± 0.1 kJ/m^2^. The impact strengths of the composites with a 20 wt% HS were lower than PP. The values of such composites were similar and ranged from 2.9 to 3.3 0.1 kJ/m^2^. This indicates a poor contribution of the reinforcements to the impact strength of the composites. Here, the MAPP content affected the impact strength of the composites with a positive correlation. When HS content was increased to 30 wt%, Impact strength values showed a higher scatter and the impact of MAPP remained positively correlated. Composites with 6 and 8 wt% MAPP contents returned impact strengths higher than the matrix. Possibly, the energy devoted to fiber pullout was enough to compensate for the contribution of the matrix. The composites with 40 wt% HS showed impact strengths higher than the matrix. Here, the effect of MAPP agreed with the un-notched results. Composites with 4 wt% of MAPP returned the highest values. The energy devoted to fiber pullout is possibly higher in the case of the composites with 4 wt% of MAPP. The composites with higher than 4 wt% MAPP contents had a stronger interface that increased the critical length of the fibers and thus restricted the number of fibers that were pulled out. Critical length is defined as:

(2) $$L_{c}=\frac{\sigma_{t}^{F}\cdot D^{F}}{2\cdot \tau }$$

where $L_{c}\;$ is the critical length, $\sigma_{t}^{F}$ is the intrinsic tensile strength of the reinforcement, $D^{F}$ is the mean diameter of the fibers and τ is the interfacial shear strength. The interfacial shear strength defines the strength of the interface and evaluates the maximum load that can be transferred from the matrix to the fiber surface. The critical length is the minimum length for a fiber to be broken due to the loads transmitted in the interface. The stronger the interface the longer the critical length. Then, fibers with a critical length will break, and shorter fibers will be pulled out. The amount of energy devoted to pullout will depend on the length of the fiber, and thus, if the critical length increases, some of the fibers that can be pulled out with a weak interface are anchored with a strong one.

**Figure 3:**
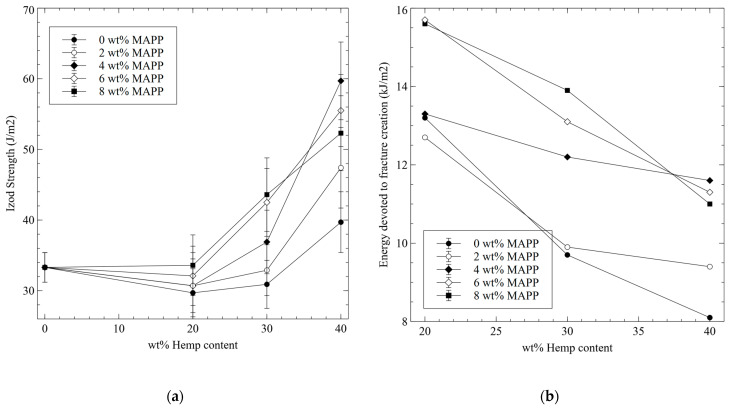
(**a**) Evolution of the impact strength for notched Charpy specimens against MAPP and HS contents; (**b**) Estimation of the evolution of the energy needed to create a critical fracture against MAPP and HS contents.

The materials were submitted to the Izod impact test to validate the values obtained from the Charpy experiments. In Figure 3, results from Izod assays are presented. 

The Izod test uses notched specimens similar to Charpy but while the Charpy impact test restricts the movement of both ends of the specimen, the Izod test only restricts one of the ends [42]. Nonetheless, the outputs are expected to be similar, not in value but in tendency. Figure 3a is very similar to Figure 2b, both in the shape and tendency of the values. Thus, the obtained values are coherent.

The impact test implies different phenomena from the original specimen to the fractured one, which needs energy. The first phenomenon is the creation of a fracture with the dimensions needed to be propagated, then, the propagation of such fracture will need more energy to skip the obstacles to such propagation. The energy necessary to break a specimen is the sum of the following parameters:

(3) $$w=w_{i}+w_{f}+w_{m}+\sum^{\;}w_{fm}$$

where w refers to the needed to break the sample, $w_{i}$ is the energy necessary to create a critical fracture, $w_{f}$ and $w_{m}$ are the energies devoted to breaking the reinforcement and the matrix, respectively. Finally, $w_{fm}$ is related to the fiber-matrix interface phenomena, like fiber pullout [43]. Notched specimens already have a critical fracture, and thus, for these specimens, $w_{i}$ takes a null value. Thus, if notched strengths are subtracted from un-notched ones, the result is the energy needed to create a critical fracture. Figure 3b shows the evolution of such energy against HS and MAPP contents.

**Figure 4:**
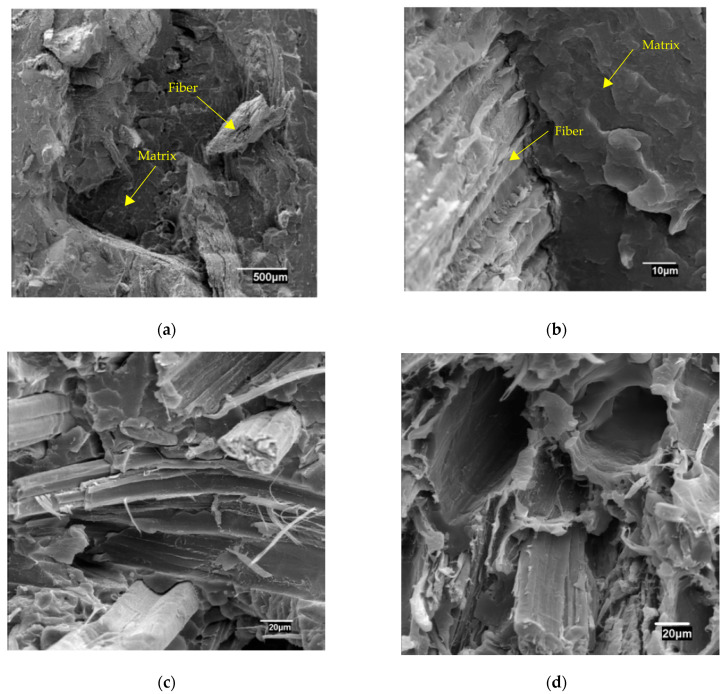
SEM micrographs of the fracture section of specimens with 40 wt% of HS; (**a**,**b**) added 8 wt% of MAPP coupling agent; (**c**,**d**) added 4% of MAPP coupling agent.

In all cases, the energy needed to create a fracture decreases with the amount of reinforcement. The slope of the regression curves is very similar for the composites adding 0, 6, and 8 wt% of MAPP. In the case of composites with a 2 wt% of MAPP, the figures show a lower slope than the aforementioned. Composites with a 4 wt% of MAPP showed the regression line with the lowest slope. From one composite to the other, the phenomena that occur during impact change, and are mainly due to the strength of the interface. Uncoupled composites will add little $w_{fm}$ due to the strength of the interface, weak in this case. Some authors agree that pullout mechanisms are one of the main contributors to the impact strength of the composites [44,45]. The contribution of the fibers will be also weak because without chemical bonding or mechanical anchoring, this contribution is highly limited. The contribution of the matrix is proportional to its content, which decreases with the amount of reinforcement. In the case of the composites with 2 wt% of MAPP, the possible increase of the strength of the interface due to the presence of MAPP makes possible some contribution of $w_{fm}$ and $w_{f}$. This explains the change in the slope of the curve with the number of fibers. Nevertheless, these contributions are not strong enough to increase noticeably the impact strength of the materials. In the case of the composites with 6 and 8 wt% of MAPP, the interface is strong and thus, the contribution of $w_{fm}$ and $w_{f}$ will be more noticeable than in the case of materials with weaker interfaces. Then, when the amount of reinforcement is increased, the interface continues increasing its strength and due to the increase in the critical length, the contribution of $w_{fm}$ is restricted. In the case of the composites with a 4 wt% of MAPP, the increase of the strength of the interface is lower and does not restrict the contribution of the fibers that pull out. Figure 4 shows the SEM micrographs of the fracture sections of composites with 40% of HS and 8 and 4 wt% of MAPP.

Figure 4a shows a few fiber pullout phenomena. The interface between the fiber and the matrix is strong and no voids are visible in such an interface (Figure 4b). This support the hypothesis of a strong interface that limits the contribution of the interfacial phenomena. Figure 4c,d show a composite with a 4 wt% of MAPP. The interface is not as strong as in the case of the composites with an 8 wt% of MAPP. There is a void region all over the fiber in Figure 4c. This allows fiber pullout and the energy needed to do so. Figure 4c shows some voids corresponding to fibers that have been pulled out. The contribution of the fibers and the interfacial regions varied noticeably with the amount of MAPP.

### 3.2. Water Uptake of PP/HS Composites

**Figure 5:**
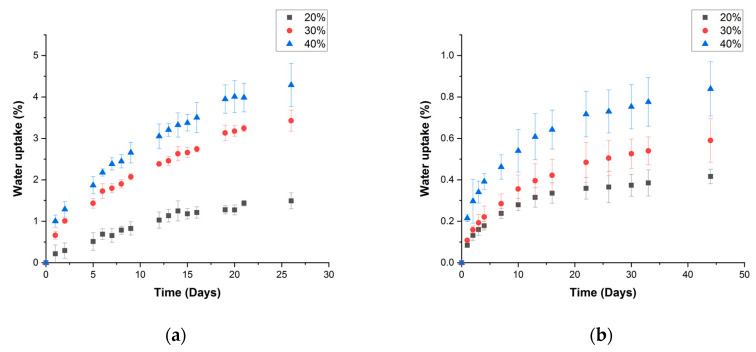
Water-absorption profiles PP composites reinforced with hemp strands under (**a**) immersion conditions and (**b**) 50% RH.

The samples of PP-HS composites coupled by 6 wt% of MAPP were submitted to the study of water uptake and a kinetic study at 23 °C through two different ways of water exposition: (1) immersion in distilled water and (2) a controlled water atmosphere of 50% RH. The profiles of water uptake are represented in Figure 5.

The data relating to PP water absorption has not been included due to its null capacity for water uptake, considered as 0, independent of the immersion time.

It is easy to observe that both mechanisms for water uptake are very slow taking into account the low thickness of the samples (3 mm). In the case of the immersion way, 25 days are needed to reach the equilibrium water uptake, being more than 40 days for the case of 50% of RH. This behavior is related to the high hydrophobic character of PP due to its long aliphatic chain based on C and H. The incorporation of hemp strands increased the water-uptake capacity of PP. This is a common phenomenon observed when polymer matrices are reinforced with reinforcements using weak compatibility [37]. The incorporation of MAPP as a coupling agent improves the compatibility between both components of the composite, but this improvement is based on points of chemical bonding, based on the ester formation between maleic anhydride and -OH groups of cellulose, not full compatibilization, remaining a big difference between the water uptake capacity of the cellulosic fibers and that of PP. This low compatibility results in huge interphases around the fibers and holes as has been observed in Figure 4d. These incompatible areas build a network of canals where water can diffuse, allowing the permeation of this fluid through the thickness of the sample. This increase of water uptake is higher when the composition in fibers is increased, reaching values of equilibrium water uptake, for formulations with 40 wt% of fibers, close to 4, in the case of immersion mechanism, and 0.8 in the case of stabilization at 50% of RH. Therefore, higher content of HS reinforcement means a higher capacity for water uptake.

**Figure 6:**
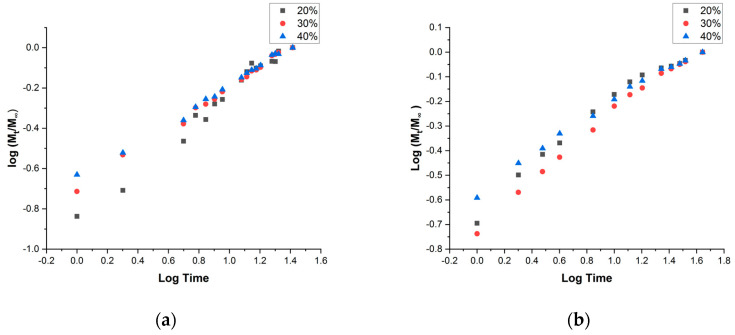
Kinetic study of PP composites reinforced with hemp strands under (**a**) immersion conditions and (**b**) at 50% RH.

Additionally, a kinetic study of the water diffusion of water through the materials has been performed. This study has been focused on the interpretation mechanism of diffusion of water considering Fick’s law (Equation (4)):

(4) $$\frac{M_{t}}{M_{\infty }}=k\cdot t^{n}$$

where $M_{t}$ and $M_{\infty }$ are the water content at time t and in the equilibrium state, respectively, and k and n are constants. Considering the water penetrating the polymer matrices, Comyn et al. [46] determined that the Fickean behavior is reached when the rate of diffusion is much lower than the mobility of the segments of the polymer. That case is characterized by a value of n coefficient close to 0.5. Figure 6 resumes a linearization of Fick’s law representing $\log\left(\frac{M_{t}}{M_{\infty }}\right)$ vs. log t for both mechanisms of the assay.

In both cases, since $\frac{M_{t}}{M_{\infty }}\leq 0.5,$ the graph tendency is linear and allows the calculation of the n coefficient. Table 2 summarizes the values of k and n values resulting from the linear adjustment of the experimental values.

In all of the cases, a linear tendency was obtained with a close to 1 value of R^2^. k value was obtained from the y-intercept and n from the slope of the tendency. The n value was close to 0.5 for every material, so the behavior of water diffusion was considered as Fickean. Espert et al. [47] considered values from 0.38 to 0.63 closer to 0.5 and consequently Fickean values. Therefore, with these results, PP reinforced with hemp strands performs an almost Fickean behavior of water uptake and the diffusion coefficient can be calculated from Equation (5) [46]:

(5)  MtM∞=4L·(Dπ)12·t12

where L is the thickness of the sample and D is the diffusion coefficient. The D values of each material are also summarized in Table 2. As it was previously mentioned, the diffusion process was very slow, but the values agree with those obtained by Espert et al. for composites of PP reinforced with different fibers such as sisal, coir, and pulped cellulose [47]. D value was increased with increasing content of fibers, except for the formulation 20% studied at 50% RH. This increase in the diffusion coefficient is related to a higher capacity of water uptake, also observed in the $\frac{M_{t}}{M_{\infty }}$ vs. time profiles and derived from the low compatibility between PP and hemp strands.

## 4. Conclusions

The influence of hemp strands as reinforcing elements on the impact resistance and the water absorption of PP composites is presented here. A complete analysis of impact tests was conducted following the samples’ geometry to allow the study of crack formation and crack propagation. The un-notched impact strength of the composites was affected by the percentage of fibers and coupling agent. There is a negative correlation between impact strength and hemp strand content for composites adding 0, 6, and 8 wt% MAPP. Instead, there is a positive correlation for composites adding 2 and 4 wt% MAPP coupling agent in the formulation. This effect has been related to the strength of the interface, the fiber dispersion, and fiber pullout mechanisms. The highest un-notched impact strengths were obtained for the materials with 20 and 30 wt% HS content coupled with 8 wt% MAPP and the composite with 40 wt% HS content coupled with 4 wt% MAPP.

Notched specimens revealed a positive correlation between fiber content and impact strength for fiber contents ranging from 30 to 40 wt%. Lower reinforcement contents returned a negative correlation. Thus, the optimum fiber contents are higher than 20 wt%. The strength of the interface has a noticeable role in the impact performance of the materials. The fiber pullout mechanism can be the main contributor to the impact strength of notched specimens.

Regarding water uptake, the incorporation of hemp strands into PP polymer increases its capacity for water absorption due to the low compatibility between both phases of the composite. This result is consistent with the increase of diffusion coefficient, which also increases with the increasing composition of hemp strands for both mechanisms of absorption, immersion, and under a controlled atmosphere.

## Figures and Tables

**Table 1:** Impact resilience of Charpy un-notched and notched, and Izod PP/HS composites against reinforcement and coupling agent contents (mean value ± standard deviation).

		Charpy Un-Notched (kJ/m^2^)	Charpy Notched (kJ/m^2^)	Izod (J/m^2^)
PP		-	3.8 ± 0.1	33.3 ± 5.9
HS (%)	MAPP (%)			
20	0	16.1 ± 1.5	2.9 ± 0.1	29.7 ± 3.4
	2	15.7 ± 1.7	3.0 ± 0.1	30.7 ± 4.7
	4	16.3 ± 1.0	3.0 ± 0.1	30.7 ± 3.8
	6	18.8 ± 1.5	3.1 ± 0.1	32.1 ± 4.2
	8	18.9 ± 1.5	3.3 ± 0.1	33.6 ± 4.3
30	0	12.7 ± 0.7	3.0 ± 0.1	30.9 ± 3.4
	2	13.1 ± 1.2	3.2 ± 0.1	32.9 ± 3.6
	4	15.8 ± 1.4	3.6 ± 0.1	36.9 ± 4.5
	6	17.2 ± 0.7	4.1 ± 0.1	42.5 ± 4.8
	8	18.2 ± 1.7	4.3 ± 0.1	43.6 ± 5.2
40	0	12.0 ± 0.7	3.9 ± 0.1	39.7 ± 4.3
	2	14.0 ± 1.2	4.6 ± 0.2	47.4 ± 5.7
	4	17.4 ± 1.4	5.8 ± 0.2	59.7 ± 5.5
	6	16.7 ± 1.6	5.4 ± 0.2	55.5 ± 5.1
	8	16.1 ± 1.7	5.1 ± 0.2	52.3 ± 5.3

**Table 2:** Values of kinetic parameters k and n, and diffusion coefficient D of PP composites reinforced with hemp strands.

Kinetic Mechanism Study: Immersion				
Formulation	k	n	R^2^	D (m^2^/s × 10^−13^)
20	0.8612	0.60	0.966	5.61
30	0.6994	0.48	0.988	8.05
40	0.6337	0.41	0.999	8.66
**Kinetic Mechanism Study: 50% RH**				
**Formulation**	**k**	**n**	**R^2^**	**D (m^2^/s × 10^−13^)**
20	0.6835	0.55	0.987	8.25
30	0.7784	0.50	0.997	7.42
40	0.5883	0.44	0.996	11.3

## Data Availability

All data explicit in the manuscript or else available at request.
